# Effects of Garlic Powder and Salt on Meat Quality and Microbial Loads of Rabbit Burgers

**DOI:** 10.3390/foods9081022

**Published:** 2020-07-31

**Authors:** Simone Mancini, Simona Mattioli, Roberta Nuvoloni, Francesca Pedonese, Alessandro Dal Bosco, Gisella Paci

**Affiliations:** 1 Department of Veterinary Sciences, University of Pisa, Viale delle Piagge 2, 56124 Pisa, Italy; roberta.nuvoloni@unipi.it (R.N.); francesca.pedonese@unipi.it (F.P.); gisella.paci@unipi.it (G.P.); 2 Department of Agricultural, Food and Environmental Science, University of Perugia, Borgo XX Giugno 74, 06100 Perugia, Italy; simona.mattioli@hotmail.it (S.M.); alessandro.dalbosco@unipg.it (A.D.B.); 3 Interdepartmental Research Center “Nutraceuticals and Food for Health”, University of Pisa, Via del Borghetto 80, 56124 Pisa, Italy

**Keywords:** spice, ingredient, colour, ready-to-cook, meat preparation

## Abstract

The aim of the research study was to evaluate the effects of a common culinary spice such as garlic powder and salt addition on the quality and microbial shelf life of rabbit meat burgers. Rabbit burgers were evaluated for pH, the colour parameters, the water holding capacity and microbial loads during storage time of seven days at 4 °C. Four different formulations of burgers (*n* = 180 in total) were tested as control samples (only meat, C), burgers with garlic powder (at 0.25%, G), burgers with salt (at 1.00%, S) and burgers with both garlic powder and salt (0.25% and 1.00%, respectively, GS). As results, it was highlighted that garlic powder and salt addition significant affected pH, water holding capacity and some colour parameters of burgers. In particular, salt affected the pH of the raw burgers, leading to lower values that partially influenced all the colour parameters with higher a* values of S burgers. The mix of garlic powder and salt (GS burgers) showed mixed effects even if more closed to the G burgers than S ones. Salt expressed its properties of binding water molecules reducing drip and cooking losses in S and GS burgers. No variations in microbial loads were highlighted in relation to the formulations. Storage time affected all the parameters, highlighting a deterioration of the burgers’ quality and an increase of the microbial loads.

## 1. Introduction

Decreasing rabbit meat consumption is spreading around the world, and the Mediterranean basin in particular, where rabbit meat was historically consumed, displays a decreasing per-capita consumption [1,2]. Other kinds of meat and the low amount of time available for cooking are affecting rabbit meat consumption, mostly among young consumers looking for more approachable kinds of food [3]. Indeed, rabbits are normally sold as pre-packed whole carcasses or cut-up (such as hind legs and loin) [4,5]. Production of meat products with rabbit meat could be a response to increase consumers’ willingness to purchase this product.

Burgers are extensively consumed as fast meals and are recognizable and well known worldwide. Burgers represent an appetizing and easy to cook protein food that does not require long cooking processes or culinary preparation. This popularity and high rate of consumption have driven the lifestyle changes that drive consumers to also prefer ready-to-cook products in meat sector.

Burgers, and in general meat products, could represent a way to reintroduce rabbit meat to daily consumption. Indeed, in the last years attention has been paid to several different types of meat used as a basis for protein burgers as this type of product could be a solution to meet the consumers’ willingness to purchase. Moreover, these combinations raise rabbit meat to a right market level of importance also in relation to its nutritional value, especially for children and the elderly [2].

Normally, burgers are sold as a ready-to-cook product, and several different recipes are available on market shelfs to attract consumers’ attention. Burgers are sold as meat mixed with spices and other ingredients among which salt is always present. Spices and herbs are used as flavours, colours and aroma enhancers and also as preservatives due to their phytochemicals [6]. Salt (NaCl, sodium chloride) due to its chemical properties, mostly the ability to reduce water activity, can also play a role as a bacteriostatic in meat products that contain a low level of salt, typically 1–3%, increasing the overall safety of the food product [7].

In this study we tested garlic (*Allium sativum* L.), a widely used spice in the Mediterranean basin, in rabbit recipes. Garlic, besides its importance as flavour carrier and sensory characteristics, can also play an import role as an antimicrobial [8,9]. In this work burgers added with salt alone and garlic powder and salt mixes were tested in order to identify more products that could reliably meet consumers’ liking. Although rabbit burgers are already on the market in some European countries where cuniculture and rabbit meat consumption have a historical tradition, such as Italy and Spain, the products have a low market penetration.

Therefore, the aim of this study was to evaluate different effects of garlic powder and salt, alone and in combinations, on burgers quality and microbial loads during refrigerated storage in order to increase our knowledge about rabbit meat products.

## 2. Materials and Methods

### 2.1. Experimental Design and Burgers Manufacture

Frozen rabbit hind legs (−20 °C, 1 month of frozen storage), derived from hybrid rabbits reared under intensive conditions (ninety day-old, 2.7 ± 0.30 kg) and fed commercial pelleted feed, were thawed for 18 h at 4 °C and then deboned. Meat was finely ground with a DN30323 meat mincer (DiNa Professional, Catania, Italy) into twelve meat batches, as previously reported [10,11]. Four different formulations (three batches per formulation) were performed as: control (C, only meat); meat supplemented with 0.25% of garlic powder (G); meat supplemented with 1.00% of salt (S); meat supplemented with 0.25% of garlic powder and 1.00% of salt (GS). Garlic powder and salt were purchased as food ingredients (garlic powder produced by Drogheria e Alimentari S.p.A., Florence, Italy, batch number: L010545; salt was sea salt type, NaCl).

Each batch was hand mixed and fifteen burgers of 100 g were formatted with a DN8097 forming machine (DiNa Professional, Catania, Italy; diameter 100 mm), for a total of forty-five burgers per formulation and 180 burgers in total. Burgers were then packaged in single Styrofoam trays, overwrapped with polyethylene film and stored raw at 4 ± 0.5 °C. At the fixed storage times (day 0, 4 and 7 of storage; D0, D4 and D7) burgers from each batch were tested as raw samples and as cooked samples.

Raw and cooked burgers were analysed at D0, D4 and D7 for the determination of the pH, the colour parameters, the water holding capacity and microbial loads (performed only on raw samples).

### 2.2. pH

The pH was measured using a pH meter (Eutech pH2700 Meter, Eutech Instruments Pte Ltd., Singapore) equipped with a XS Sensor Standard S7 (XS Sensor, Modena, Italy) and an automatic temperature compensator. pH meter was calibrated before each session with buffer solutions at pH 4.01 and 7.01 (HI7004L and HI7007L Hanna Instruments, Padova, Italy).

### 2.3. Colour

Colour was expressed as L*(lightness), a* (redness), and b* (yellowness) according to the CIElab system [12]. Colour parameters were measured using a Minolta CR300 chroma meter (Minolta, Osaka, Japan) with an aperture size of 8 mm, illuminant D65 and incidence angle of 0°. Before each session, the colorimeter was calibrated with a white tile (L* = 98.14, a* = −0.23 and b* = 1.89). Each data point was the mean of three replications measured on the surface of the burgers at randomly selected locations. Chroma (C*) and Hue angle (h*) were calculated as function of a* and b* following the formulas:

$$C^{*}=\sqrt{\left(a^{{*}^{2}}+b^{{*}^{2}}\right)}$$

$$h^{*}=\tan^{-1}\left(\frac{b^{*}}{a^{*}}\right)$$

The total colour difference (ΔE) was calculated as proposed by Sharma and Bala [13] between two different formulations at the same storage time or between two different storage times for the same formulation following the formula:

$${\Delta E}_{\alpha -\beta }=\sqrt{{\left(L_{\alpha }^{*}-L_{\beta }^{*}\right)}^{2}+{\left(a_{\alpha }^{*}-a_{\beta }^{*}\right)}^{2}+{\left(b_{\alpha }^{*}-b_{\beta }^{*}\right)}^{2}}$$

where α and β subscripts of L*, a* and b* referred to two different formulations at the same storage time or two different storage times for the same formulation. Cooking effect on colour were determined for each F at each storage time (ST). As proposed by Sharma and Bala [13], the threshold of a human noticeable difference was fixed at 2.3 points.

### 2.4. Water Holding Capacity

Drip loss was calculated as proposed by Lundström and Malmfors [14] within the F between D0-D4 and D0-D7. Cooking loss was calculated as percentage of the decrease of weight before and after cooking in a preheated oven at 163 °C to an internal temperature of 71 °C and were turned every 4 min to prevent excess surface crust formation [15].

### 2.5. Microbial Quantifications

Ten grams of sample were aseptically removed and homogenised in a Stomacher 400 Circulator Lab Blender (Seward, Worthing, UK) with 90 mL of 0.1% peptone salt solution. Further serial dilutions were made in the same diluent and used for standard plate enumerations.

Total aerobic mesophilic and psychrotrophic bacteria were determined on Plate Count Agar (pour plate method) with incubation at 30 °C for 72 h, and 7 °C for 10 days, respectively; Enterobacteriaceae on Violet Red Bile Glucose Agar at 37 °C for 24 h; *Escherichia coli* on Tryptone Bile X-Glucuronide Medium (TBX) at 44 °C for 24 h, lactic acid bacteria on MRS Agar (pour plate method) in anaerobiosis (Anaerogen 2.5L) at 30 °C for 72 h; *Brochothrix thermosphacta* on Streptomycin Thallous Acetate (STA) agar with STA selective supplement at 25 °C for 48 h; *Pseudomonas* spp. on Pseudomonas Agar base with CFC supplement at 25 °C for 72 h; yeasts and moulds on Yeast Extract Glucose Chloramphenicol Agar (pour plates method) after incubation at 25 °C for 5 days. Where not specified, spread plate method was used. All cultural media and supplements were from Oxoid (Basingstoke, UK). The bacterial counts were expressed as log Colony-Forming Units (CFU) per gram of sample.

### 2.6. Statistical Analysis

The effects of the formulation (F), of the storage time (ST) and their interaction (F × ST) on the burger parameters were analysed through a two-way ANOVA using the R software Version 1.2.5019 (R Core Team, The R Foundation for Statistical Computing, Vienna, Austria) [16]. The significance level was set at 5% (statistically significant for *p* < 0.05), and if statistical significance was found, the differences were assessed using Tukey’s test (*p* < 0.05). When the interaction F × ST was not significant the results are reported as the mean of the fixed effects F and ST; the variability is expressed as root mean square error (RMSE).

## 3. Results and Discussion

Results of the pH, the colour parameters and the water holding capacity of the raw burgers are reported in Table 1. The formulation (F) significatively effected all the tested parameters. Furthermore, also storage time influenced quite all the parameters, indeed, only L* and b* coordinates did not show significant differences for ST (*p* = 0.099 and = 0.066, respectively). The addition of salt lead to lower pH values in average, as evidenced by the pH of S and GS formulations in relation to C and G burgers (*p* = 0.002). These differences in pH values might played a role in the colour values; indeed L* of S and GS burgers were lower than C and G ones (*p* < 0.001), as pH and lightness are linked by a negative correlation. Additions of garlic powder modified the redness (a*) and yellowness (b*) coordinates (*p* < 0.001 and *p* = 0.013, respectively). Natural pigments in garlic powder lead to a pale-yellow raw product than when processed turn to a strong yellow colour turning more vivid. Due to degradation processes the garlic pigments could produce a green-yellow tone [17,18,19]. Garlic natural pigments showed their effect in G and GS burgers with the reduction of redness value, as well as an increase of yellowness value. Additions of ingredients, both salt and garlic powder and its mix, decreased the chroma index in relation to the C burgers, beside S and GS burgers showed a greater decrement in chroma as salt addition affected negatively both a* and b* coordinates (*p* < 0.001). On the other hand, garlic induced an increase in h* leading to light-yellow burgers in colour due to the increase of b* value at the expense of a* value (*p* < 0.001).

During storage time a slight increase in pH was revealed at D7 (*p* = 0.002), that might be imputable to an alkalinisation of meat resulting from an increase in ammoniacal nitrogen levels and to the degradation of proteins and amino acids by Gram-negative bacteria [20,21,22]. Also the reduction of a* and C* and the increase of h* during storage time (*p* < 0.001 for all indexes) could be ascribed to bacteria metabolism actions and due to the formation of metmyoglobin produced by myoglobin oxidation [23,24].

Water holding capacity was affected principally from salt addition as both S and GS burgers showed lowest drip and cooking losses (*p* = 0.016 and *p* < 0.001, respectively), indeed salt due to its chemical properties contributes to water and fat binding in meat products and this property is enhanced by mincing processing [25,26,27]. As expected during storage time drip loss increased due to the natural water release, furthermore, the loss of water by the raw product during the storage lead to day 7 at more dry samples with a consequence of a lowest cooking loss.

Cooking flattered the formulation differences, with statistical evidence only on L* and h* parameters (Table 2). After cooking the presence of salt in the samples affected the L* value with lower values of S ang GS than C and G samples (*p* < 0.001), following the trend reported in raw samples. Even if a* and b* coordinates were not affected by F (*p* = 0.065 and *p* = 0.278, respectively) the h* index revealed a difference in colour between C and the other formulation that appeared lighter in colours (*p* = 0.004). During storage time a rise in b* value was highlighted (*p* < 0.001) as degradation of the pale pink colour of rabbit meat and the formation of yellowness complex. As consequence of b* value rise also both C* and h* increased their values meaning a lighter vivid yellowness samples at D7 than D0 (*p* < 0.001 and *p* = 0.032, respectively).

Cooking might affect several chemical, physical, and even biological characteristics of the products. As burger require a cooking section to be eaten it is important how this final step is performed in order to maintain the chemical and nutritional properties added via formulation and physical properties related to sensory acceptance by consumers [28,29].

Colour differences (ΔEs) within F between ST are reported in Table 3; colour differences (ΔEs) within ST between F are reported in Table 4. All the F changed in a noticeable way the overall colour during the 7 days of storage (D0–D7). The addition of the sole garlic powder induced a strong variation colour after 4 days as reported by the ΔE value of D0–D4 period. That might be related to the rapid oxidation of the garlic compounds and the formation of a green-yellow hue that mitigate the pink rabbit meat colour. This modification in raw burgers affected also the ΔE between cooked burgers at D0 versus D4 as G formulation was the only one to reported values over the threshold of 2.3 points. On the contrary C, GS S cooked burgers showed higher variation in colour between D4 and D7.

Colour differences at the same storage time between F highlighted that salt addition strongly changed the burgers’ colour at D0 in both raw and cooked samples, reaching high ΔEs between S and GS in relation to C and G formulations. This tendency was maintained also at D4 even though also G burgers increase their colour distances from the control burgers. After cooking no noticeable differences was reported between S and GS burgers, countering the garlic effect on colour. After 7 days of storage the cooked burgers showed the lowest ΔE values between the formulations highlighting that the oxidation process occurred by the time affected all the samples inducing a general colour variation.

In Table 5 are reported the microbial loads of the raw burgers. No statistical differences were highlighted for the F main factor; thus, no effect of salt or garlic addition was evidenced on the microbial load. *Escherichia coli* and *Brochothrix thermosphacta* were not detected in the burgers both in relation to the formulation and the storage time. All the detected bacterial loads increased during storage time (*p* < 0.001 for all the parameters), mostly with differences between each fixed day of analysis. Only the total aerobic psychrotrophic bacteria showed to reach the highest value at D4 and to maintain it at D7.

Spices and other ingredients added into minced meat products, such as burgers, might affect in different ways the growth of microorganisms. Turmeric and ginger powders showed a bacteriostatic effect against several different bacteria in rabbit burgers stored at 4 °C [20,23]. Similarly, pork burgers/patties supplemented with different plant products, such as passion fruit co-products and tea or grape extracts, showed a lower bacterial growth than the respective control treatment [30,31,32]. Ingredients’ activity against microorganisms’ growth might be also related to the physical form and technological transformations, to the employed concentrations or to the meat used. Indeed, Sallam et al. [33] reported activities against aerobic plate count of fresh garlic (30 g/kg) and garlic powder (9 g/kg) added in chicken sausages stored at 3 °C up to 21 days. Also Aydin et al. [34] reported activities of fresh garlic (10%) in ground beef refrigerated for 24 h in terms of total aerobic mesophilic bacteria and coliform bacteria.

## 4. Conclusions

The additions of garlic powder and salt to rabbit meat could bring several characteristics modifications to burgers along with culinary perceptions. Both garlic powder and salt also played a role in the colour changes in relation to the storage time. No effects on the microbial loads suggest that higher concentrations of garlic powder or salt are needed if their use is to be also intended as bacteriostatic additives. Different garlic products such as fresh minced or extracts could produce higher/lower beneficial effects, thus further studies are needed to better the potential application of this spice and how addition of salt and/or garlic could affect burgers’ flavour and consumers’ acceptance.

## Figures and Tables

**Table 1:** pH, colour parameters and water holding capacity of raw rabbit burgers.

Item	Formulation (F)				Storage Time (ST, Days)			*p*-Value			RMSE
	C	G	S	GS	D0	D4	D7	F	ST	F × ST	
pH	5.97 ^a^	5.96 ^a^	5.87 ^b^	5.86 ^b^	5.89 ^y^	5.89 ^y^	5.97 ^x^	0.002	0.002	0.360	0.058
L*	57.24 ^a^	57.65 ^a^	51.31 ^b^	51.04 ^b^	55.38	53.80	53.75	<0.001	0.099	0.861	2.769
a*	5.86 ^a^	4.62 ^b^	5.53 ^a^	4.09 ^b^	5.57 ^x^	5.34 ^x^	3.97 ^y^	<0.001	<0.001	0.343	0.759
b*	6.07 ^b^	7.43 ^a^	5.93 ^b^	6.41 ^ab^	7.04	5.97	6.37	0.013	0.066	0.252	0.938
C*	10.34 ^a^	8.83 ^ab^	8.29 ^b^	7.71 ^b^	10.19 ^x^	8.10 ^y^	8.07 ^y^	<0.001	<0.001	0.234	1.209
h*	40.70 ^b^	58.46 ^a^	48.19 ^b^	58.70 ^a^	44.22 ^y^	49.34 ^y^	60.97 ^x^	<0.001	<0.001	0.224	6.250
Drip loss%	0.63 ^a^	0.77 ^a^	0.40 ^b^	0.44 ^b^	0.00 ^z^	0.73 ^y^	0.95 ^x^	0.016	<0.001	0.316	0.835
Cooking loss%	24.33 ^a^	22.00 ^a^	17.83 ^b^	16.15 ^b^	21.07 ^x^	21.17 ^x^	17.99 ^y^	<0.001	0.032	0.828	0.024

C: control; G: control + 0.25% of garlic powder; S: control + 1% of salt; GS: control + 0.25% of garlic powder + 1% salt. ^a,b^ Different letters in the same row indicate significant differences at *p* < 0.05 for F. ^x,y,z^ Different letters in the same row indicate significant differences at *p* < 0.05 for ST.

**Table 2:** pH and colour parameters of cooked rabbit burgers.

Item	Formulation (F)				Storage Time (ST, Days)			*p*-Value			RMSE
	C	G	S	GS	D0	D4	D7	F	ST	F × ST	
pH	6.13	6.11	6.07	6.06	6.10	6.07	6.11	0.218	0.053	0.893	0.102
L*	69.70 ^a^	70.60 ^a^	66.43 ^b^	65.31 ^b^	67.76	68.61	67.66	<0.001	0.452	0.201	2.278
a*	7.19	6.48	6.09	6.18	6.29	6.21	6.95	0.065	0.567	0.084	0.959
b*	14.77	14.27	15.23	16.14	14.09 ^y^	14.58 ^y^	16.64 ^x^	0.278	<0.001	0.522	1.341
C*	16.43	15.70	16.42	17.29	15.44 ^y^	15.86 ^y^	18.09 ^x^	0.567	<0.001	0.291	1.436
H*	64.09 ^b^	65.98 ^a^	68.02 ^a^	68.97 ^a^	65.90 ^y^	66.97 ^x^	67.42 ^x^	0.004	0.032	0.102	2.563

C: control; G: control + 0.25% of garlic powder; S: control + 1% of salt; GS: control + 0.25% of garlic powder + 1% salt. ^a,b^ Different letters in the same row indicate significant differences at *p* < 0.05 for F. ^x,y^ Different letters in the same row indicate significant differences at *p* < 0.05 for ST.

**Table 3:** Colour differences (ΔE) within Formulation (F) between Storage Time (ST).

	Storage Time (ST, Days)								
	Raw Samples			Cooked Samples			Raw–Cooked Samples		
**Formulation (F)**	D0–D4	D4–D7	D0–D7	D0–D4	D4–D7	D0–D7	D0	D4	D7
C	2.21	3.09 *	5.18 *	0.97	3.38 *	4.32 *	13.65 *	14.93 *	17.42 *
G	4.66 *	2.51 *	3.57 *	3.69 *	6.96 *	3.58 *	12.63 *	18.39 *	14.51 *
GS	2.72 *	2.57 *	5.18 *	2.78 *	3.06 *	4.50 *	15.76 *	19.30 *	20.05 *
S	2.48 *	2.24	4.33 *	1.96	3.11 *	2.37 *	15.27 *	16.57 *	20.34 *

* Value over the threshold (2.3 points) with a noticeable difference in colour between the samples. C: control; G: control + 0.25% of garlic powder; S: control + 1% of salt; GS: control + 0.25% of garlic powder + 1% salt.

**Table 4:** Colour differences (ΔE) within Storage Time (ST) between Formulation (F).

	Storage Time (ST, Days)											
	D0				D4				D7			
**Formulation (F)**	C	G	S	GS	C	G	S	GS	C	G	S	GS
C		*2.81* *	*8.21* *	*7.21* *		*4.84* *	*7.29* *	*7.39* *		*4.94* *	*5.37* *	*4.94* *
G	**0.67**		*8.27* *	*6.64* *	**4.03** *		*6.15* *	*5.27* *	**0.51**		*7.02* *	*8.10* *
S	**5.77** *	**5.40** *		*1.73*	**2.67** *	**6.26** *		*3.70* *	**2.84** *	**2.41** *		*1.15*
GS	**5.34** *	**4.82** *	**2.25**		**6.21** *	**9.93** *	**1.88**		**3.53** *	**3.06** *	**1.68**	

* Value over the threshold (2.3 points) with a noticeable difference in colour between the samples. C: control; G: control + 0.25% of garlic powder; S: control + 1% of salt; GS: control + 0.25% of garlic powder + 1% salt. In the columns of ΔEs within ST between F the values in italic or bold refer respectively to raw and cooked samples.

**Table 5:** Microbial determinations on raw rabbit burgers.

Item	Formulation (F)				Storage Time (ST, Days)			*p*-Value			RMSE
	C	G	S	GS	D0	D4	D7	F	ST	F × ST	
Enterobacteriaceae	3.80	3.94	3.77	3.94	1.59 ^z^	3.96 ^y^	6.04 ^x^	0.824	<0.001	0.691	0.445
*Pseudomonas* spp.	5.41	5.31	4.91	4.96	2.23 ^z^	5.73 ^y^	7.48 ^x^	0.507	<0.001	0.984	0.748
Lactic acid bacteria	3.49	3.79	3.42	3.99	2.21 ^z^	3.36 ^y^	5.44 ^x^	0.633	<0.001	0.969	0.923
Yeast and moulds	3.69	3.44	3.19	3.09	1.99 ^z^	3.34 ^y^	4.73 ^x^	0.355	<0.001	0.665	0.686
Total aerobic mesophilic bacteria	6.62	6.49	6.05	6.21	4.22 ^z^	6.73 ^y^	8.08 ^x^	0.293	<0.001	0.692	0.603
Total aerobic psychrotrophic bacteria	6.29	5.71	5.95	5.53	3.61 ^y^	6.42 ^x^	7.58 ^x^	0.703	<0.001	0.794	1.299

C: control; G: control + 0.25% of garlic powder; S: control + 1% of salt; GS: control + 0.25% of garlic powder + 1% salt. ^x,y,z^ Different letters in the same row indicate significant differences at *p* < 0.05 for ST.
